# The Effects of Epistemic Trust and Social Trust on Public Acceptance of Genetically Modified Food: An Empirical Study from China

**DOI:** 10.3390/ijerph17207700

**Published:** 2020-10-21

**Authors:** Longji Hu, Rongjin Liu, Wei Zhang, Tian Zhang

**Affiliations:** School of Public Administration, Central China Normal University, Wuhan 430079, China; hulongji@mail.ccnu.edu.cn (L.H.); liurongjing@mails.ccnu.edu.cn (R.L.); zhangtian@163.com (T.Z.)

**Keywords:** epistemic trust, risk perception, genetically modified food, public acceptance, partial least squares structural equation modeling

## Abstract

Most studies exploring the public acceptance of genetically modified food (GMF) are based on social trust and the establishment of a causal model. The underlying premise is that social trust indirectly affects public acceptance of GMF through perceived risks and perceived benefits. The object of social trust is trust in people, organizations, and institutions. Different from the social trust, epistemic trust refers to people’s trust in scientific knowledge behind the technology of concern. It has been shown that epistemic trust, like social trust, is also an important factor that affects the public perception of applicable risks and benefits. Therefore, it is necessary to incorporate epistemic trust into the causal model to derive a more complete explanation of public acceptance. However, such work has not been conducted to date. The causal model proposed in this paper integrated epistemic trust and social trust and divided social trust into trust in public organizations and trust in industrial organizations. A representative questionnaire survey (N = 1091) was conducted with Chinese adults. The model was analyzed by the partial least squares structural equation modeling (PLS-SEM) method. Three major findings were obtained: First, epistemic trust is an important antecedent of perceived risks and perceived benefits and exerts a significant indirect effect on the acceptance of GMF. Secondly, trust in industrial organizations negatively impacts perceived risks, while trust in public organizations positively impacts perceived benefits. Thirdly, contrary to the common opinion, trust in industrial organizations did not exert a significant direct effect on perceived benefits, and trust in public organizations did not demonstrate a significant direct effect on perceived risks. Therefore, trust in industrial organizations and trust in public organizations utilize different influence paths on GMF acceptance. This study enriches the understanding of the influence path of trust with regard to the acceptance of emerging technologies and is of great significance to relevant risk-management practices.

## 1. Introduction

Genetically modified food (GMF) offers a significant technological advance in modern agriculture as well as enormous social and economic benefits [1]. Like any other new food technology in history, the safety of GMF has been a source of anxiety, uncertainty, controversy, and low acceptance since GMF entered the market. Previous studies have shown that consumers from Japan, the European Union (EU), and the United States (US) maintain low acceptance of GMF [2,3,4,5,6,7,8,9,10]. In this regard, China is no exception [11]. A survey by Cui and Shoemaker (2018) showed that the percentage of the Chinese public adverse to GMF is as high as 41.4% [12]. The commercialization of GMF as well as the decision-making of stakeholders related to food industries (e.g., governmental policymakers, farmers, and agro-biotechnology enterprises) strongly depends on its public acceptance [13,14,15]. Given the importance of predicting public acceptance of GMF, much scholarly attention has been paid to explore the factors that affect the public acceptance of GMF [16,17,18,19,20,21,22].

Among these factors, scholars are most interested in the following three: perceived risks, perceived benefits, and trust [13,17]. Siegrist (2000, 1999) proposed a causal model to explain the acceptance of gene technology, which identified perceived benefits, perceived risks, and trust as the three most important factors to affect public acceptance. Moreover, the perceived benefits and perceived risks directly influence acceptance, while trust imposes an indirect influence on acceptance through perceived risks and perceived benefits [23,24]. This model has been accepted as the basic model for the study of the acceptability of emerging technologies and is widely used in several technical fields.

Trust in the casual model of public acceptance mostly refers to social trust [25]. Social rust is a psychological state, which comprises the intention to accept vulnerability based on positive expectations of the intentions or behavior of another [26,27]. The object of social trust is trust in people, organizations, and institutions. Trust is a multi-type concept. Except social trust, epistemic trust is also one kind of trust that has an important impact on perceived risks. Epistemic trust refers to people’s trust in scientific knowledge behind the technology of concern [28]. Some studies have shown that cognitive trust is an important factor in perceived risk, which is more negatively correlated with perceived risk than social trust [29]. However, with the exception of some recent studies, most causal models of public acceptance rarely consider epistemic trust. For example, Hakim et al. (2020) did not explicitly mention the concept of epistemic trust but included the content of epistemic trust in the measurement items of social trust [18].

In addition, social trust is also a multi-type concept. According to the types of trustees, some scholars divided social trust into trust in public organizations (e.g., government regulators, public research institutions) and trust in industrial organizations (e.g., farmers and agro-biotechnology enterprises) [30]. Several studies have found that these two types of social trust have different antecedents. The level of trust in public organizations depends on their abilities and competence, and the level of trust in industrial organizations depends on their intentions, honesty, and integrity [31,32]. At the same time, some studies found that public trust in these two types of trustees differ: people have more trust in public organizations than in industrial organizations [33]. Therefore, it is necessary to divide the social trust into trust in industrial organizations and trust in public organizations; then, the impact of these two types of social trust on public acceptance of GMF can be appropriately explored.

This study complements previous studies in two aspects: first, the concept of epistemic trust is introduced as an indirect factor that affects public acceptance; second, different from most previous studies, which, as outlined, regarded trust in industrial organizations and trust in public organizations as a single construct, this study treats both types of trust as two different variables.

All in all, some studies have found that epistemic trust is an important factor affecting the public risk perception; trust in public organizations and trust in industrial organizations should be treated as two different variables. By integrating epistemic trust, trust in public organizations, and trust in industrial organizations into the causal model, the impact of trust on public acceptance of GMF can be appropriately explored. However, such work has not been conducted to date. As such, this study is aimed at integrating epistemic trust, trust in public organizations, and trust in industrial organizations into one model and analyzing their impacts on public acceptance of GMF.

## 2. Research Hypotheses and Framework

Public acceptance of GMF (ACC) is defined as the willingness of the public to buy and their intention to use GMF [34,35]. As technical barriers disappear, a critical factor for promoting the deployment of GMF is whether people will be willing to buy and use the resulting products and enjoy the benefits of this technology [36,37].

The role ACC plays in the implementation of food technology induces a number of important research questions for social scientists, such as “which factors determine whether a particular food technology will be accepted or rejected?” A wide range of social–psychological factors influencing ACC has already been explored. Among these, three factors (social trust, perceived benefits, and perceived risks) have attracted the most scholarly attention [17,38]. To explain ACC, Siegrist (2000, 1999) proposed a causal model for the relationship among these three factors [23,24]. In that causal model, Siegrist argued that acceptance of this new technology was determined by both perceived risks and perceived benefits and that social trust exerted a strong indirect influence on acceptance through perceived risks and benefits.

This causal model has been widely used to explain public acceptance in a variety of technological domains, such as gene technology [18,39,40,41], financial technology [42,43], nanotechnology [44,45,46,47,48,49], renewable energy [32,50,51,52], unmanned aircraft [53], and automated driving technology [54,55]. The present study is also based on Siegrist’s causal model.

**Figure 1:**
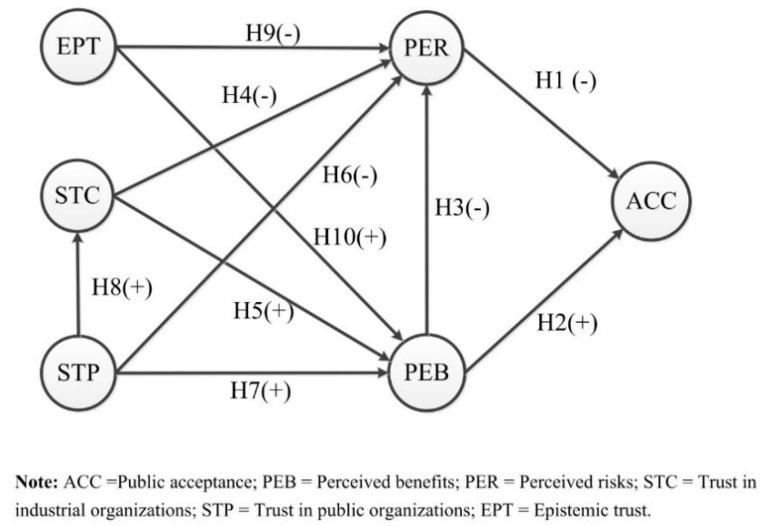
The concept model.

The present study proposed a research framework to systematically examine the relationship among these various types of trust, perceived risks/benefits, and public acceptance of GMF. Figure 1 illustrates the proposed framework.

### 2.1. Perceived Risks and Benefits

Perceived risk is an individual’s impression or interpretation of the uncertainty and potential negative consequences related to an object that is perceived as a threat [56,57]. Different people will interpret the same objective facts differently and thus form different subjective cognition. It has been shown that because of the discrepancy and biases in individual cognition, not only the individuals but also the perceived risks that have been assessed by experts can differ widely [58]. Moreover, for the same risk, different individuals will have completely different perceived outcomes. Perceived benefit is an individual’s perception of the benefits a certain technology or product can provide [59]. Benefits are what individuals pursue when they accept a certain technology to buy a certain product, which are thus reflections of the value these individuals want to obtain [60].

In the case of GMF, perceived risks include unknown long-term effects, side effects on human health, and both environmental and social problems [61,62,63]. Perceived benefits revolve around environmental issues, especially in relation to the reduction of energy and chemical inputs, high yields and diversity, lower food prices, and longer shelf life [64,65].

In general, food products have been regarded as low-involvement purchases that require only limited decision-making [66]. Perceived risks have no explanatory power unless they exceed a specific threshold [67]. Most surveys showed that the risk perceptions of the public toward GMF are high across the world [11,68,69,70,71,72,73]. In the context of GMF, perceived risks may be a key factor that determines ACC as perceived benefits [16,74,75]. Most studies and reviews have concluded that perceived benefits positively influence the acceptance of GMF. In contrast, perceived risks are considered to impose negative impacts. There have been good reviews on the relationship among perceived risks, perceived benefits, and GMF acceptance; please refer to Frewer, Lynn J., et al. (2013), Bearth A. and M. Siegrist (2016), and Machado Nardi, V.A., et al. (2020) [13,19,34].

Based on the above, the following related hypotheses are proposed:

**Hypothesis H1** **(H1).** *Public’s perceived risks of GMF will negatively affect their ACC.*

**Hypothesis H2** **(H2).** *Public’s perceived benefits of GMF will positively affect their ACC.*

Most research suggested that risk and benefit perceptions were not independent but rather inversely related [76,77]. This suggests that people do not judge risks and benefits independently, as is done in scientific risk–benefit appraisals, but rather they intuitively weigh risks and benefits against each other. When the risks people thus perceive are high, they perceive that the benefits that could be obtained are low, and vice visa. Following Siegrist (1999), this further proposes that perceived benefits influence perceived risks [24]. The public has little capacity and knowledge to properly evaluate the risks associated with GMF; however, its benefits are tangible and concrete, and people consequently are more experienced about the associated benefits [39,78]. Therefore, for normal people, it is much easier to assess the benefits associated with GMF than to assess its risks. Attributing relatively high benefits and high risks to GMF would produce cognitive dissonance. It would not be surprising if altering the level of perceived risks reduced this dissonance. Following this argument, it is more plausible that perceived benefits influence perceived risks than the opposite.

**Hypothesis H3** **(H3).** *Public’s perceived benefits of GMF will negatively affect their perceived risks.*

### 2.2. Social Trust

Trust is a psychological state, which comprises the intention to accept vulnerability based on positive expectations of the intentions or behavior of another [26]. In this definition, the object is trust in people, organizations, and institutions. This type of trust is also referred to as social trust [25,27,79].

Most people do not possess elaborated knowledge about GMF [20,80,81]. One way to cope with this lack of knowledge and capacities is to rely on others to evaluate and manage an associated hazard. Relevant actors are the producers of GMF, regulating organizations (e.g., governmental organizations), research institutions working in the field of GMF, and independent non-governmental organizations [82]. Therefore, social trust plays a dominant role in such circumstances. Research in the domain of GMF showed that people who trust responsible actors attributed more benefits and fewer risks to GMF. Thus, social trust indirectly impacts the acceptance of GMF [37,83,84].

Social trust is a multi-type construct. Peters, Covello, and McCallum (1997) divided social trust into trust in the industry, trust in the government, and trust in citizen groups [30]. They showed that the trust in the industry was related to public perceptions of concern and care on the part of this industry (e.g., the trustee’s intentions, honesty, and integrity). The trust in the government was related to perceptions of commitment and perceptions of knowledge and expertise (e.g., trustee’s abilities and competence). The trust in citizen groups was related to perceptions of knowledge and expertise (e.g., trustee’s abilities and competence). Maeda and Miyahara (2003) conducted a similar study in Japan. They obtained the same results about trust in government and industry but not for citizen groups because the social positions of citizen groups are not yet stable in Japan [85]. Thus, it can be seen that different types of social trust have different antecedents. The antecedents of trust in public organizations lie in the trustee’s abilities and competence, and the antecedents of trust in industrial organizations lie in the trustee’s intentions, honesty, and integrity. Additionally, Lang and Hallman (2005) and Terwel et al. (2009) found that the trust level of the public in these different organizations is also different. Generally, the trust level of public organizations is higher than that of industrial organizations [33,86]. Finally, Maeda and Miyahara (2003) found that the trusts in both government and industry were negatively related to risk perception [85].

Based on these studies, each type of social trust has different antecedents, and the public assigns different levels to every type of trust. Therefore, it is essential to distinguish between these various types of social trust and divide them into two differential constructs. In this way, we can have a deeper understanding of the influence mechanism of social trust on public acceptance of GMF.

In China, the social positions of citizen groups are not yet stable because their social positions have only recently started to be established. Following Maeda and Miyahara (2003), this study inferred that citizen groups are not well trusted by the people in China [85]. Consequently, citizen groups are not seen as relevant agents in the domain of GMF and were not included in the investigation. This study distinguished between trust in the industrial organizations which form the industry and trust in public organizations. Since previous studies showed that social trust, as one single construct, exerts significant influences on the perceived benefits and the perceived risks, it is reasonable to assume that both types of social trust might influence perceived benefits and perceived risks in the same way.

However, these two types of social trust are not independent. The responsibility of public institutions is to supervise the effective implementation of relevant risk policies and regulations by industrial organizations. López-Navarro, Llorens-Monzonís, and Tortosa-Edo (2013) argued that the more effective public institutions perform their supervision responsibilities, the higher the public’s confidence in the industrial organizations’ compliance with relevant risk control measures will be [14]. Consequently, trust in public organizations will directly and positively impact the trust in industrial organizations. In fact, evidence supports this argument.

Following these arguments, the following set of hypotheses was developed:

**Hypothesis H4** **(H4).** *Higher trust in public organizations will decrease the perceived risks of GMF.*

**Hypothesis H5** **(H5).** *Higher trust in public organizations will increase the perceived benefits of GMF.*

**Hypothesis H6** **(H6).** *Higher trust in industrial organizations will decrease the perceived risks of GMF.*

**Hypothesis H7** **(H7).** *Higher trust in industrial organizations will increase the perceived benefits of GMF.*

**Hypothesis H8** **(H8).** *Trust in public organizations will directly and positively influence trust in industrial organizations.*

### 2.3. Epistemic Trust

In her study of the Storuman (Sweden) referendum on siting a local high-level nuclear waste repository, Drottz-Sjöberg (1996) found that even though the social trust may exist, the public may still reject a siting proposal. People may well trust the technical expertise of an industry or an agency in the area of technology of concern, but they may still reject the technology [87]. It is clear that this kind of rejection could not be explained by lower social trust. Such a contradiction is common in society. Sjöberg (2001, 1999) argued that the reason for such contradictions is that people distrust the sufficient development of the scientific basis of the technology of concern, assuming that current scientific knowledge has not completely assessed the (negative) effects of the technology [28,88].

Sjöberg (2001) thus introduced the concept of epistemic trust to represent people’s trust in scientific knowledge behind the technology of concern [28]. Additionally, research showed that social trust is fairly marginal when it comes to account for perceived risks [89,90]. Follow-up work supported the notion that epistemic risk is an important factor for perceived risks, which was even more strongly negatively related to perceived risks than social trust [25,29,52,91,92,93,94,95,96].

Based on these findings, the following hypotheses were proposed:

**Hypothesis H9** **(H9).** *Epistemic trust will negatively affect perceived risks.*

**Hypothesis H10** **(H10).** *Epistemic trust will positively affect perceived benefits.*

## 3. Methods

### 3.1. Sample

Data were collected through self-reported, structured questionnaires. The questionnaire was developed in Chinese and was submitted to a panel of five experts at one of the key universities in Central China for content validity evaluation. Of these five experts, two work at the Department of Biology and three at the School of Public Management. The panel approved both the issue list and question format and suggested revisions to clarify questions so that the general public could fully understand the questions asked and answer them. Before the formal survey, a pilot test was conducted. In summary, 50 randomly selected individual participants (representing the public, and including both 20 undergraduates and 30 ordinary people) were interviewed individually. During the test, respondents were asked whether they could clearly understand the questions and felt comfortable answering them. According to their feedback, changes were implemented with regard to wording, expressions, and grammar to improve the questionnaire’s clarity, accuracy, flow, and validity.

The questionnaire contained four parts: The first section was the screening question “Have you heard of genetically modified food?” The respondent need not continue to fill in the questionnaire if his or her answer was “no”. The second section asked for socio-demographic information including gender, age, educational background, and income. The third section focused on the public’s acceptance of GMF. The last part inquired about perceived risks/benefits level with regard to GMF, social trust in different objects, and epistemic trust.

The survey followed a stratified sampling. First, to account for geographical differences and to maximize representativeness, we choose eight provinces from the east (Zhejiang), south (Guangdong), west (Sichuan, Xizang, and Xinjiang), north (Hebei), northeast (Jilin), and middle (Hubei) regions in China. Two higher-income and another two lower-income counties were randomly selected in each province, resulting in 32 counties. Next, 4–6 city communities or villages were randomly selected from each county, resulting in 150 city communities or villages. Finally, 7–10 households were randomly approached in each of the city communities or villages, resulting in a total sample of 1200 observations. In June 2019, through public recruitment, 100 university students were recruited as interviewers at Central China Normal University. The home addresses of the university students were located in the 32 counties selected above; there are 3–4 interviewers in each county. Face-to-face interviews were conducted by university students during July and September 2019.

A total of 1200 paper-based questionnaires were distributed. Finally, 1168 paper-based questionnaires were thus collected, resulting in 1091 valid questionnaires after eliminating those with clerical errors or contradictions; the effective questionnaire recovery rate reached 93.41%.

### 3.2. Measures

The measurement scale used in this study contained six constructs and twenty-two items, which are based on several scales in relevant studies (see Table 1 for specific relevant studies) that offer high reliability and validity. Peculiarities of the Chinese language and culture were considered during the translation. A minor modification in the wording was made to suit Chinese peculiarities. The subjects were asked to indicate their agreement or disagreement with the statements provided, using a seven-point Likert scale. Table 1 provides detailed scale items for the constructed variables.

To assess ACC, a 5-item measure was used that was developed by Siegrist (1999, 2000) and Zhang (2017) and represented people’s willingness to buy GMF under different specified circumstances [23,24,97]. The first two items were adapted from Zhang (2017), and the remaining three items were adapted from Michael Siegrist (1999, 2000). Examples of items are “Whenever possible I avoid buying GMF” (1 = strongly disagree; 7 = strongly agree) (reverse-coded) and “Would you like to buy genetically modified food?” (1 = strongly unwilling; 7 = strongly willing).

To assess the perceived benefits of GMF, the 4-item measure was used that was developed by Sjöberg (2005) and Ghoochani et al. (2016) [96,98]. The items therein reflect the social and environmental benefits of GMF that are typically mentioned by experts and news media. Items that represent the economic benefits of GMF (e.g., lower price, reduction of production cost, and increased profit) were not included, because previous studies found that most of the public assumed that these economic benefits were only obtained by the corporations in the domain of GMF [96]. An example of these items is “Transgenic technology can increase crop yields and feed more people.” (1 = strongly disagree; 7 = strongly agree).

To assess the perceived risks of GMF, respondents indicated their agreement with the 5-items developed by Sjöberg (2005), Chen M.-F. (2008), Ghoochani et al. (2016), and Zhang (2019) [37,96,98,99]. Two items reflect the possible harm of GMF to human health, and the other two items reflect the possible harm of GMF to the environment. Examples of items are “Eating genetically modified food will lead to infertility.” and “Planting genetically modified crops will have a negative impact on the environment.” (1 = strongly disagree; 7 = strongly agree).

Trust in industrial organizations: Although previous studies have shown that social trust contains multiple components, Lang and Hallman (2005) showed that these components were highly correlated and would converge on a common factor [86]. Therefore, the public’s social trust in different objects can be measured in a specific holistic way. Based on this argument, the trust in industrial organizations was measured via the trust in various industrial organizations [39,100]. Participants were asked, “How much trust do you have in the following institutions: (1) food corporations, (2) agricultural corporations, (3) pharmaceutical corporations?” Participants had to indicate their level of trust on a 7-point-scale, ranging from no trust at all to a very high level of trust (1 = not at all; 7 = very much).

Trust in public organizations was measured via trust in various public organizations [39,96]. Participants were asked, “How much trust do you have in the following institutions: (1) National Food Administration, (2) Public research institutions in the domain of GMF, (3) National Institute of Public Health?” Participants had to indicate their level of trust on a 7-point-scale, ranging from no trust at all to a very high level of trust (1 = not at all; 7 = very much).

To measure epistemic trust, respondents indicated their agreement with the three items that were adopted from Sjöberg (2005) and that have been used elsewhere [94,96]. An example of such items is “There could be negative side effects of GMF unknown for scientific knowledge today” (1 = strongly disagree; 7 = strongly agree).

### 3.3. Analysis Method

Descriptive statistics and exploratory factor analysis of all questionnaire items were performed in a preliminary study by IBM SPSS Statistics (IBM, Armonk, NY, USA). In contrast to covariance-based structural equation methods (CB-SEM), partial least squares structural equation modeling (PLS-SEM) is the appropriate analytical tool in this case because it imposes minimal demands on measurement scales, sample size, and residual distributions [101]. PLS-SEM was used to test the reliability and validity of both the measurement model and the structural model. Smart PLS version 3.2.4 [102] was used to run the PLS–SEM analysis. A bootstrapping procedure of 5000 resamples was used to generate t-statistics and standard errors [103].

The process of evaluation of the results of the PLS-SEM involves two steps. In step 1, the assessment of the measurement model is conducted. When measurement quality is confirmed, the structural model evaluation is conducted in step 2 [104].

For the assessment of the measurement model, we start by examining the indicator loadings. Loadings above 0.70 indicate that the construct explains more than 50% of the indicator’s variance, demonstrating that the indicator exhibits a satisfactory degree of reliability. The constructs’ internal consistency reliability was assessed. For Cronbach’s alpha (**α**), higher values indicate higher levels of internal consistency reliability. Results between 0.70 and 0.95 represent “satisfactory to good” reliability levels [104]. Composite reliability (CR) measures internal consistency reliability that assumes the same thresholds. Results between 0.70 and 0.95 represent “satisfactory to good” reliability levels [104].

Next, the convergent validity was calculated, which is the extent to which a construct converges in its indicators by explaining the items’ variance. Convergent validity is assessed by the average variance extracted (AVE) across all items associated with a particular construct and is also referred to as communality. An acceptable threshold for the AVE is 0.50 or higher. This level or higher indicates that, on average, the construct explains (more than) 50% of the variance of its items [104].

The last stage is to assess discriminant validity. This analysis reveals to which extent a construct is empirically distinct from other constructs both in terms of how much it correlates with other constructs and how distinctly the indicators represent only this single construct. Discriminant validity assessment in PLS-SEM involves analyzing Henseler et al.’s (2015) heterotrait–monotrait ratio (HTMT) of correlations [105]. The suggested threshold is a value of 0.85 when the path model included constructs that are conceptually very similar.

For the assessment of the structural model, we start by testing the proposed research hypothesis about the causal relationship between latent variables by evaluating the significance of the path coefficients. A path coefficient is regarded as significant if the p-value is below the pre-defined α-level.

Next, for the significant path coefficients, it makes sense to quantify how substantial they are, which can be accomplished by assessing their effect size ***f*^2^**. ***f*^2^** values above 0.35, 0.15, and 0.02 can be regarded as strong, moderate, and weak, respectively [106].

Finally, the multicollinearity of the structural model is examined. The index used is the variance inflation factor (VIF). If VIF is less than 5, it can be considered that there is no serious multicollinearity problem in the structural model [104].

## 4. Results

### 4.1. Descriptive Analysis

A total of 1091 individuals with a mean age (standard deviation) of 32.93 (14.31) years were enrolled in this study. The sample did not originate from strict random sampling, and therefore, the representativeness of the sample had to be evaluated. To do so, a χ^2^ test was applied to ensure that the sample in this study is representative of the entire population. Table 2 presents the characteristics of the sample and the results of the χ^2^ test, which indicate that the sample could represent the Chinese population roughly (*p* ≥ 0.05).

### 4.2. Assessment of Measurement Model

Cronbach’s alpha (**α**) and composite reliability (CR) were above 0.70 and each AVE (Average Variance Extracted) was above 0.50 (see Table 3), indicating that the measurements were reliable and that the latent construct can account for at least 50% of the variance within items. As shown in Table 3, the loadings are within an acceptable range and the t-values indicate that they are significant at the 0.001 level.

The discriminant validity of the constructs was evaluated using the approaches recommended by the heterotrait–monotrait ratio (HTMT) of correlations [105]. If the HTMT is significantly smaller than 0.85, this is evidence of sufficient discriminant validity. The results in Table 4 suggest that all constructs had acceptable discriminant validity.

### 4.3. Common Method Bias

All data were collected by using the discussed survey method; therefore, common method bias may affect the validity of this research.

First, the data set was assessed using Harman’s one-factor test to identify any potential common method biases [107]. The danger of common method bias is high if a single factor accounts for more than 50% of the variance [108]. Evidence of common method bias exists when a general construct accounts for the majority of the covariance among all constructs. A principal component factor analysis was performed and the results excluded the potential threat of common methods bias. The first (and largest) factor accounted for 27.930% and none of the general factors accounted for more than 50% of the variance, indicating that common method bias was not a serious problem in the data set.

Second, following Liang et al. (2007), the PLS model included a common method factor whose indicators included all principal constructs’ indicators [109]. Each calculated indicator’s variances could be substantively explained by the principal construct and by the method. The results demonstrate that the average substantively explained variance of the indicators was 0.665, while the average method-based variance was 0.015. The ratio of substantive variance to method-based variance is 44:1. In addition, all method-based factor loadings are non-significant. Given the small magnitude and insignificance of method-based variance, according to Liang et al. (2007), this indicated that the method is unlikely to be a serious concern for this study.

### 4.4. Assessment of Structural Model

Model fit was evaluated by examining the goodness of fit (GoF) [110] and the standardized root mean square residual (SRMR) [111]. GoF was 0.475, which exceeded the cut-off value of 0.36 for a large effect size [110]. SRMR evaluates the discrepancy between an observed correlation matrix and a predicted correlation matrix. The calculated SRMR was 0.039, which remained below the recommended threshold of 0.08 [111]. Thus, the PLS path model achieved an appropriate overall fit.

**Figure 2:**
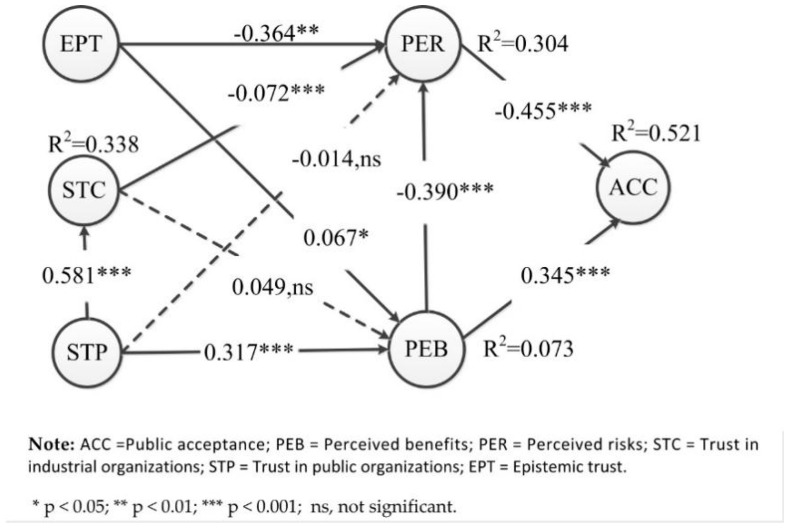
Result of partial least squares (PLS) analysis for structural model.

Figure 2 and Table 5 presents the estimates obtained via PLS analysis. R^2^ indicates the amount of variance explained by a model [112]. To evaluate the full model, R^2^ values were calculated for ACC. The R^2^ value of 0.521 indicated that the model explains a substantial amount of variance for ACC.

Perceived risks demonstrated a direct and statistically significant negative relationship with ACC (*β* = −0.455, *p* < 0.001). Individuals who perceived more risks were less likely to accept GMF, thus supporting Hypothesis 1.

Perceived benefits demonstrated a direct and statistically significant positive relationship with ACC (*β* = 0.345, *p* < 0.001). Individuals who perceived more benefits were more likely to acceptance of GMF, thus supporting Hypothesis 2.

As shown in Table 4, perceived risks (***f*^2^** = 0.303) exerted a greater influence than perceived benefits (***f*^2^** = 0.196) for affecting public acceptance of GMF.

Perceived benefits demonstrated a direct and statistically significant negative relationship with perceived risks (*β* = −0.390, *p* < 0.001). Individuals who perceived more benefits were more likely to perceive fewer risks, thus supporting Hypothesis 3.

Higher trust in industrial organizations demonstrated a direct and statistically significant negative relationship with perceived risks (PER) (*β* = −0.072, *p* < 0.05). The more people trusted industrial organizations, the fewer their perceived risks, thus supporting Hypothesis 4.

Higher trust in industrial organizations did not demonstrate a direct, statistically significant relationship with perceived benefits, thus Hypothesis 5 was not supported. In this sample, individuals with high trust in an industrial organization were not likely to perceive more benefits.

With regard to Hypothesis 6, Figure 2 shows that the higher trust in public organizations → perceived risks link was not significant, thus failing to confirm that people’s trust in public organizations affects their risk perception.

As shown in Figure 2, the positive higher trust in public organizations → perceived benefits link was significant (*β* = 0.317, *p* < 0.001), thus supporting Hypotheses 7. Individuals with high trust in public organizations were likely to perceive more benefits.

With regard to Hypothesis 8, Figure 2 shows that the higher trust in public organizations → higher trust in industrial organizations link was significant (β = 0.581, *p* < 0.001). The more people trust public organizations, the more they will trust industrial organizations.

Figure 2 also shows that the negative epistemic trust → perceived risks link was significant (β = −0.364, *p* < 0.001) and the positive epistemic trust → perceived benefits link was weakly significant (β = 0.067, *p* =0.069), but it was only significant at *p* = 0.10, hence, supporting Hypotheses 9 and 10. Individuals with high trust in the science behind GMF were likely to perceive more benefits and fewer risks.

Trust in public organization (***f*^2^** = 0.073) had a stronger influence than epistemic trust (***f*^2^** = 0.006) on explaining perceived benefits. The impact of trust in the industrial organization on perceived benefits was small (***f*^2^** = 0.003).

The effects of epistemic trust and perceived benefits had an influence on explaining perceived risks that were similar (***f*^2^** = 0.181 and 0.195, respectively). Trust in industrial organizations on perceived risks and the impact of higher trust in public organizations were similar (***f*^2^** = 0.006 and 0.001, respectively).

In tests of multicollinearity, the variance inflation factor (VIF) values were calculated for all constructs. All VIF values remained well below the acceptable threshold of 5.0 (ranging from 1.000 to 1.707), as shown in Table 6 [113].

## 5. Discussion

### 5.1. Theoretical Implications

By integrating social trust [23,24] and epistemic trust [25,28,114] in risk perception research, this study proposes an extended model to explain GMF acceptance. The model specifies the relationship among social trust, epistemic trust, perceived risks, perceived benefits, and GMF acceptance. The model was tested with survey data of China. This study yielded three major findings: First, epistemic trust is an important antecedent to perceived risks and perceived benefits and exerts a significant indirect effect on GMF acceptance. Secondly, higher trust in industrial organizations exerts a negative impact on perceived risks, and higher trust in public organizations exerts a positive impact on perceived benefits. Therefore, higher trust in industrial organizations and higher trust in public organizations utilize different influence paths toward GMF acceptance. Thirdly, in contrast to common opinion, higher trust in industrial organizations did not exert a significant direct effect on perceived benefits, and higher trust in public organizations did not exert a significant direct effect on perceived risks. This study enriches the knowledge on the influence path of trust on the acceptance of emerging technology and has significance for risk management practice.

Consistent with prior research, these findings suggest that perceived risks and perceived benefits are two important factors that influence GMF acceptance. By demonstrating that perceived benefits have a statistically significant, negative relationship with perceived risks, this study supported that risk and benefit perceptions are not independent but rather inversely related [76,77].

More importantly, consistent with Sjöberg [25,28], the findings of the present study illustrate that epistemic trust is an important factor in GMF acceptance by exerting a direct positive effect on perceived benefits and a negative effect on perceived risks. Given that research has identified epistemic trust as an important antecedent to perceived risks and perceived benefits, future research should not omit this construct.

With regard to social trust, these findings also suggest that it is important to distinguish between trust in industrial organizations and trust in public organizations. When both types of trust are treated jointly in the same model, differences were found with respect to the GMF acceptance relationship, depending on the type of trust. Trust in industrial organizations in the domain of GMF exerts a negative impact on perceived risks, and trust in public organizations exerts a positive impact on perceived benefits. In contrast to the hypothesized relationships, higher trust in industrial organizations did not exert a significant direct effect on perceived benefits, and higher trust in public organizations did not exert a significant direct effect on perceived risks. On the one hand, these findings confirm that it is necessary to distinguish between higher trust in industrial organizations and higher trust in public organizations when operationalizing the social trust measurement. Furthermore, it is important to consider both types of trust separately as two different constructs. On the other hand, the results show that the influence paths of both types of trust on the GMF acceptance differ.

These findings are consistent with the results of a number of recent studies. When they distinguished between trust in companies and trust in public institutions, López-Navarro, Llorens-Monzonís, and Tortosa-Edo (2013) showed that trust in companies negatively affected citizens’ health risk perception [14]. Moreover, trust in public institutions did not exert a direct and significant effect in the context of a petrochemical industrial complex. However, their study did not include the relationship between both types of trust and perceived benefits; therefore, a complete comparative analysis could not be conducted. Peters, Covello, and McCallum (1997), as well as Maeda and Miyahara (2003), showed that the component (or dimension) of trust in the government (i.e., public organizations) is a form of competency trust, while the component (or dimension) of trust in the industry (i.e., industrial organizations) is a form of goodwill trust [30,85]. In the context of nuclear power plants (NPPs) in China, Xiao, Liu, and Feldman (2017) showed that goodwill trust improves the acceptance of NPPs by decreasing the risk perception of the public [32]; however, competence trust improves the acceptance of NPPs by increasing the benefit perception. The authors showed that the associations between goodwill trust and benefit perception, as well as competence trust and risk perception, were not significant. Considering the results of Peters, Covello, and McCallum (1997) as well as Maeda and Miyahara (2003), the results of the present study are highly consistent with the results of Xiao, Liu, and Feldman (2017) [30,32,85].

Why does higher trust in industrial organizations only affect the perceived risks, while higher trust in public organizations only affects perceived benefits? The literature provides no explanation for this question. The risks of GMF are mainly related to industrial activities; therefore, industrial organizations are the first to bear the responsibility for managing the risks inherent to the industrial activity, which they developed [14]. Therefore, trust in industrial organizations is directly related to reducing the public’s perception of the risks associated with GMF. At the same time, according to the loss aversion principle, most people apply asymmetric sensitivity to gains and losses, and the pain in the face of loss is perceived as far stronger than the pleasure in the face of gain [115]. For most people, the primary goal of decision-making is to avoid risks and losses. Only when risks are controlled and when safety is guaranteed, most people further consider the benefits. The duties of public organizations in the domain of GMF are associated with their regulatory activities as well as their functions of authorizing and monitoring relevant industrial activities. The component (or dimension) of trust in public organizations is a form of competency trust. When people have a high level of trust in public organizations, they assume that public organizations have the ability to perform their duties effectively. Under the premise that loss is avoided, the public will pay more attention to the relevant benefits of GMF. Therefore, trust in public organizations exerts a significant positive impact on perceived benefits.

### 5.2. Policy Implications

These results offer relevant implications for the practitioner. Scientific knowledge contains inherent uncertainty. In this sense, it is rational for the public to criticize and doubt scientific knowledge. However, such criticism and doubt should be based on relevant evidence and facts. In China, most people lack the scientific knowledge to understand transgene technology and biology in general [116]; therefore, it is difficult for them to make a reasonable judgment on the scientific evidence about GMF. It is easy for the lay public to distort the uncertainties of scientific knowledge behind GMF, which further reduces their willingness to accept GMF. To overcome this issue, the government can use a variety of popular science activities to improve the scientific literacy of the public, enhance their epistemic trust, and ultimately improve GMF acceptance.

The results of this study may be useful for industrial organizations in the domain of GMF in relation to their behavior. Industrial organizations ought to be concerned about the level of trust received by the public in response to all items representing higher trust in industrial organizations. All average values of the items were lower than the middle value of the scale (see Table 3). Given this fact, and considering that the component of higher trust in industrial organizations is goodwill trust, industrial organizations should pay more attention to public interests. Moreover, they should improve the transparency of their risk control information to enhance goodwill trust.

The findings for public organizations are of concern: contrary to the case of higher trust in industrial organizations, the average values of all items representing higher trust in public organizations were higher than the middle value of the scale (see Table 3). Although public trust in public organizations exceeds that in industrial organizations, the absolute level of higher trust in public organizations is low, and the average values of all items representing higher trust in public organizations remain below 5 (see Table 3). Therefore, and considering that the component of higher trust in public organizations is competence trust, public organizations need to properly execute their regulatory function with regard to the risk management of GMF. This will further enhance the public’s trust in their capabilities and increase the perceived benefits in the public.

Both industrial organizations and public organizations in the domain of GMF should focus their efforts on prompting public trust, reducing perceived risks, and improving benefit perception, which will ultimately improve GMF acceptance. Generating and maintaining trust often constitutes the primary goal of industrial communication policies.

### 5.3. Limitations

Although the findings of this study provide meaningful implications for both researchers and practitioners, a number of limitations apply. On the one hand, this study used cross-section data on research methods and its acceptance intention took the place of actual acceptance behavior as the explained variable. While the current studies mostly used this method, considering that public acceptance of GMF is a dynamic process, future studies should consider longitudinal dynamic tracking surveys. Moreover, the GMF acceptance of the public should be compared for different periods to better reflect the changing regularity of this acceptance. On the other hand, this study focused on the influence of trust factors on perceived benefits, perceived risks, and public acceptance without considering other factors. Consequently, the explanatory power of a number of the variables remains limited, especially for perceived benefits (*R*^2^ = 0.073). Future studies should expand the model by adding further important influencing factors.

## Figures and Tables

**Table 1:** Measures used in the study.

Construct	Items	Source
Public acceptance (ACC)	(ACC1) Would you like to buy genetically modified food?	[23,24,97]
	(ACC2) Would you like to buy this kind of food if the product trademark indicates that it contains genetically modified ingredients?	
	(ACC3) Whenever possible I avoid buying GMF (reversed scoring).	
	(ACC4) Compared with ordinary food, genetically modified food has a longer shelf life. Would you choose to buy because of this point?	
Perceived benefits (PEB)	(PEB1) Overall, GM food technology is useful for society.	[96,98]
	(PEB2) Transgenic technology can increase crop yields and feed more people.	
	(PEB3) GMF creates a higher quality of life; it is a great technological advancement.	
	(PEB4) Genetically modified foods will eventually be accepted by the majority of people.	
Perceived risks (PER)	(PER1) Overall, GMF can be dangerous to people.	[37,96,98,99]
	(PER2) Eating genetically modified food will lead to infertility.	
	(PER3) Eating genetically modified food will change the genes of us or future generations.	
	(PER4) The production of genetically modified food will destroy the diversity of animals and plants.	
	(PER5) Planting genetically modified crops will have a negative impact on the environment.	
Trust in industrial organizations (STC)	(STC1) food corporation.	[39,100]
	(STC2) agricultural corporation.	
	(STC3) pharmaceutical corporation.	
Trust in public organizations (STP)	(STP1) National Food Administration.	[39,96]
	(STP2) public research institution in the domain of GMF.	
	(STP3) National Institute of Public Health.	
Epistemic trust (EPT)	(EPT1) There could be negative side effects of GMF unknown for scientific knowledge today.	[94,96]
	(EPT2) Scientific knowledge about GMF is probably still incomplete	
	(EPT3) Researchers behind GMF technology are hardly aware of all consequences of what they create.	

**Table 2:** Descriptive statistics of the sample data.

Characteristic	Classification	Number	Sample (%)	Population (%) *	χ^2^ Test (*p*-Value)
Gender	Male	483	44.3	51.2	0.982 (0.322)
	Female	608	55.7	48.8	
Age	15–29 years old and below	523	47.9	42.9	0.902 (0.637)
	30–50 years old	447	41.0	42.3	
	51 years old and above	121	11.1	14.8	
Type of Habitat	Rural inhabitant	585	53.6	55.9	0.081 (0.776)
	Urban inhabitant	506	46.4	44.1	
Education background	Primary education	183	16.8	27.7	4.744 (0.192)
	Junior high school	427	39.1	40.6	
	High school (including technical secondary school)	254	23.3	17.5	
	College degree and above (including junior College)	227	20.8	14.2	
Monthly income (Chinese Yuan)	<3000	843	77.3%	No available	
	3001–5000	204	18.7%	No available	
	>5001	44	4.0%	No available	

* Source: http://www.stats.gov.cn/tjsj/zxfb/201604/t20160420_1346151.html.

**Table 3:** Confirmatory factor analysis results.

Construct	Mean(SD)	Item	Mean	SD	Loading	P	α	CR	AVE
ACC	3.682(1.481)	ACC1	3.432	2.071	0.827	0.000	0.804	0.872	0.640
		ACC2	3.372	1.631	0.840	0.000			
		ACC3	4.813	1.642	0.800	0.000			
		ACC4	3.112	2.142	0.701	0.000			
EPT	2.852(1.161)	EPT1	2.531	1.379	0.801	0.000	0.777	0.857	0.668
		EPT2	2.742	1.382	0.773	0.000			
		EPT3	3.301	1.421	0.874	0.000			
PEB	4.479(1.232)	PEB1	4.711	1.501	0.858	0.000	0.816	0.879	0.645
		PEB2	4.751	1.558	0.768	0.000			
		PEB3	4.282	1.589	0.853	0.000			
		PEB4	4.169	1.519	0.725	0.000			
PER	3.887(1.129)	PER1	3.801	1.561	0.820	0.000	0.802	0.862	0.558
		PER2	3.682	1.468	0.794	0.000			
		PER3	3.551	1.659	0.761	0.000			
		PER4	4.151	1.492	0.711	0.000			
		PER5	4.282	1.371	0.634	0.000			
STC	4.078(1.292)	STC1	3.850	1.451	0.864	0.000	0.884	0.928	0.811
		STC2	4.253	1.401	0.921	0.000			
		STC3	4.161	1.471	0.916	0.000			
STP	5.258(1.191)	STP1	5.661	1.382	0.800	0.000	0.765	0.864	0.680
		STP2	5.162	1.471	0.822	0.000			
		STP3	4.961	1.460	0.850	0.000			

Note: ACC =Public acceptance; EPT = Epistemic trust; PEB = Perceived benefits; PER = Perceived risks; STC = Trust in industrial organizations; STP = Trust in public organizations.

**Table 4:** Discriminant validity (heterotrait–monotrait ratio (HTMT)).

	ACC	EPT	PEB	PER	STC	STP
ACC						
EPT	0.239					
PEB	0.668	0.154				
PER	0.755	0.436	0.466			
STC	0.316	0.120	0.270	0.183		
STP	0.305	0.326	0.422	0.137	0.694	

Note: ACC =Public acceptance; PEB = Perceived benefits; PER = Perceived risks; STC = Trust in industrial organizations; STP = Trust in public organizations; EPT = Epistemic trust.

**Table 5:** Causal relationships.

Path	Path Coefficients	$\mathrm{Effect}\;\mathrm{Size}\;(\mathrm{Cohen}’s\;f^{2})$	t-Value	*p* Values	Hypothesis Check
PER -> ACC	−0.455	0.303(medium -large)	22.473	0.000	H1 (Supported)
PEB -> ACC	0.345	0.196(medium)	15.260	0.000	H2 (Supported)
PEB -> PER	−0.390	0.195(medium)	13.463	0.000	H3 (Supported)
STC -> PER	−0.072	0.006(small)	2.147	0.032	H4 (Supported)
STC -> PEB	0.049	0.003(small)	1.303	0.193	H5 (Not supported)
STP -> PEB	0.317	0.073(small–medium)	8.454	0.000	H6 (Supported)
STP -> PER	−0.014	0.001(small)	0.410	0.682	H7 (Not supported)
STP -> STC	0.581	0.513(large)	26.571	0.000	H8 (Supported)
EPT -> PER	−0.364	0.181(medium)	12.917	0.000	H9 (Supported)
EPT -> PEB	0.067	0.006(small)	1.820	0.069	H10 (Supported)

Note: ACC =Public acceptance; PEB = Perceived benefits; PER = Perceived risks; STC = Trust in industrial organizations; STP = Trust in public organizations; EPT = Epistemic trust.

**Table 6:** Variance inflation factors (VIFs) of the constructs.

	ACC	PEB	PER	STC
EPT		1.057	1.062	
PEB	1.271		1.133	
PER	1.433			
STC		1.541	1.543	
STP		1.593	1.707	1.000

Note: ACC =Public acceptance; PEB = Perceived benefits; PER = Perceived risks; STC = Trust in industrial organizations; STP = Trust in public organizations; EPT = Epistemic trust.
